# Mediation effect of knowledge management on the impact of IT capability on firm performance: exploring the moderating role of organization culture management

**DOI:** 10.3389/fpsyg.2024.1344330

**Published:** 2024-09-18

**Authors:** Weiwei Wu, Xue Li, Bowornsintuchon Surangkana

**Affiliations:** School of Management, Harbin Institute of Technology, Harbin, Heilongjiang, China

**Keywords:** information technology capability, organizational culture management, firm performance, knowledge stock, knowledge process

## Abstract

**Introduction:**

With the development of the digital economy, a multitude of firms have embarked on the path of digital transformation through information technology (IT). Scholars have called for attention to the mediating or moderating mechanisms of IT capability on firm performance. This study argues that further exploration is needed regarding the relationship between IT capability and firm performance.

**Methods:**

This study obtained questionnaire data from 152 IT senior managers of randomly selected manufacturing firms. The data was used to empirically test the proposed hypotheses using hierarchical regression analysis.

**Results:**

The results showed that IT capability has a positive effect on firm performance directly and indirectly via knowledge stock and knowledge process. Moreover, organizational culture management moderates the relationship between knowledge stock/knowledge process, and firm performance.

**Discussion:**

This study proposes the “resources-knowledge-performance” mechanism, which sheds light on the “black box” of how IT capability affects firm performance. This enriches the research on knowledge from different perspectives and the the research on organizational culture by discussing the moderating role of organizational culture management in the relationship between knowledge stock/process and firm performance. Our research also has important managerial implications to firm.

## 1 Introduction

With the development of the digital economy, a multitude of firms have embarked on the path of digital transformation through information technology (IT). The ability of firms to integrate other resources with information resources is referred to as information technology capability (Chae et al., 2018). In recent years, the impact of IT capability on firm performance has been a focal point of scholarly attention. Some studies posit a positive effect of IT capability on firm performance (Hao and Song, 2016; Li and Chan, 2019). However, there are also inquiries that challenge this notion, suggesting that IT capability might have no significant impact or even a negative effect on firm performance (Chae et al., 2014; Rahman and Zhao, 2020; Mamonov and Peterson, 2021). Some studies have investigated the mediating mechanisms through which IT capability influences firm performance, such as innovativeness, dynamic capabilities, business process, and supply chain management capabilities (Peng et al., 2016; Mikalef et al., 2020; Marchiori et al., 2022). Furthermore, certain studies have examined the boundary conditions of the relationship between IT capability and firm performance, including industry, operations capability, and intelligence-infused operations capability (Chae et al., 2018; Wang et al., 2023; Zhang H. et al., 2023). The advancement of business intelligence and the widespread use of analytic tools that enhance IT capability make it more crucial than ever to understand if a firm with stronger IT capability is in the digital era (Rahman and Zhao, 2020). Based on the existing research debates and the needs arising from the digital era, scholars have called for attention to the mediating or moderating mechanisms of IT capability on firm performance (Mamonov and Peterson, 2021; Wang et al., 2023). This study argues that further exploration is needed regarding the relationship between IT capability and firm performance.

IT capability plays a crucial role in facilitating knowledge acquisition, knowledge conversion, and knowledge application (Braojos et al., 2020), which in turn impacts firm performance. Drawing on knowledge management theory, researchers have diverged from a singular focus on knowledge as an object or process (Hsu and Shen, 2005). The object and process views explain how knowledge is generated through the cyclic interaction between knowledge stock and knowledge process flow. However, the existing research has yet to fully integrate both the object and process views of knowledge in investigating the mediating role of knowledge management capability in the relationship between IT capability and firm performance. This limitation hinders a comprehensive understanding of the mechanisms through which knowledge management capability operates. Therefore, this study aims to explore the significant role of knowledge management capability as a mediating factor in the process of enhancing firm performance driven by IT capability by considering both the knowledge stock and knowledge process flow in knowledge management capability.

The theory of knowledge management posits the importance of organizational culture in facilitating successful knowledge creation, sharing, and utilization, which ultimately leads to innovation (Ajmal and Helo, 2010) and how it facilitates or hinders this process. Organizational culture encompasses the assumptions, values, norms, and tangible signs (artifacts) within an organization. The processes of creating and sharing knowledge are intangible and rely on voluntary cooperation. Therefore, it is essential for organizations to foster a culture that encourages and supports the generation and dissemination of knowledge (Gray and Densten, 2005; Martinez-Caro et al., 2020). This study suggests that the level of organizational culture management may influence the relationship between knowledge management capability and firm performance.

Based on the resource-based theory and knowledge management theory, this study integrates IT capability, knowledge management capability, organizational culture management, and firm performance into the research framework. It establishes a theoretical model based on the “resources-knowledge-performance” logic and conducts empirical research on the paths of the impact of IT capability, knowledge management capability, and firm performance. It also explores the moderating role of organizational culture management in the relationship between knowledge management capability and firm performance.

This study makes three contributions: First, it contributes to the research on the mediating mechanism of IT capabilities on firm performance. Previous literature has explored the effects of knowledge management capability on firm performance, but there has been limited research that combines IT and the process and object of knowledge management to investigate the mechanisms and paths of firm performance. This study proposes the “resources-knowledge-performance” mechanism and finds that knowledge stock and knowledge flow partially mediate the relationship between IT capabilities and firm performance. It sheds light on the “black box” of how IT capability affects firm performance. Second, this study contributes to the research on knowledge management. Based on the process and object view of knowledge, this study includes knowledge stock and knowledge process flows in the research. This enriches the research on knowledge from different perspectives. Third, this study contributes to the research on organizational culture. It discusses the moderating role of organizational culture management in the relationship between knowledge stock/process and firm performance. This study provides new insights into how operations capability enhances firm performance and contributes to the research on institutional theory and knowledge management theory.

## 2 Theoretical background and hypothesis development

### 2.1 Theoretical basis

#### 2.1.1 Resource-based view in information system

The resource-based view (RBV) posits that a firm’s success depends on its ability to acquire and leverage unique and valuable resources and capabilities (Lioukas et al., 2016; Andrade-Rojas et al., 2021). Within this framework, IT capability is regarded as a unique organizational resource that can help firms process information more effectively, improve efficiency, and enhance innovation. High levels of IT capability, particularly those that incorporate organization-specific knowledge, culture, and processes into IT systems, are often difficult for competitors to replicate, thereby constituting a firm’s unique advantage. IT capability is not just a single technology or system but a platform that integrates and coordinates other resources and capabilities. This integration capability enables IT to play a critical role in facilitating cross-departmental collaboration, fostering knowledge sharing, and supporting complex decision-making processes (Pan et al., 2015; Elia et al., 2021). In brief, the RBV highlights the strategic importance of IT capability as a unique resource that contributes to firm performance.

#### 2.1.2 Knowledge management theory

The theory of knowledge management (KM) refers to a theoretical framework that aims to enhance organizational innovation capability, learning capability, and competitiveness through the effective organization and utilization of knowledge resources (Gold et al., 2001; Lee et al., 2020). When knowledge is considered an object, the emphasis is on accumulating and overseeing knowledge stock. When seen as a process, the focus shifts to the dynamics of knowledge creation, dissemination, and sharing. The knowledge process underlines the necessity for organizations to utilize their existing knowledge to foster new insights. The concept of knowledge stock draws attention to the importance of absorptive capacity (Yongping et al., 2011), which is heavily reliant on the learner’s pre-existing knowledge base. Therefore, effective knowledge management capability within organizations demands attention to both the accrual of knowledge stock and the knowledge process (Gray and Densten, 2005).

The theory of knowledge management posits the importance of organizational culture in facilitating successful knowledge creation, sharing, and utilization, which ultimately leads to innovation (Ajmal and Helo, 2010). The processes of creating and sharing knowledge are intangible and rely on voluntary cooperation. Therefore, it is essential for organizations to foster a culture that encourages and supports the generation and dissemination of knowledge (Gray and Densten, 2005; Martinez-Caro et al., 2020).

### 2.2 It capability and firm performance

The ability of firms to integrate other resources with information resources is referred to as information technology capability (Chae et al., 2018). From an operational perspective, the higher the level of IT capability, the more it can promote the intelligent manufacturing of production departments, optimize production processes, and improve production efficiency (Mamonov and Peterson, 2021). A higher level of IT capability can also help sales departments establish electronic sales platforms, allowing for product sales without the time and location constraints (Dong and Yang, 2019). Additionally, the warehouse and logistics departments can dynamically adjust inventory levels and transportation times using IT. As IT capability increases, the inventory turnover efficiency of the firm will also improve (Chae et al., 2014). Therefore, IT capability can enhance operational performance. Based on this, the following research hypotheses are proposed:

H0a: IT capability has a positive impact on productivity.

From a financial perspective, by utilizing IT and online platforms, firms can better understand consumer preferences and their changes. With a strong IT capability, firms can improve existing products or develop new ones in a more timely and accurate manner (Kmieciak et al., 2012; Chae et al., 2018). This enables them to gain a larger market share or explore new product markets, ultimately achieving profit growth by expanding the customer base (Marchiori et al., 2022). Additionally, IT capability can facilitate data analysis and decision-making, allowing firms to make more informed financial decisions and allocate resources effectively (Zhang H. et al., 2023). Hence, IT capability can improve financial performance. Based on this assertion, the following research hypothesis is proposed:

H0b: IT capability has a positive impact on profitability.

### 2.3 It capability, knowledge stock, and knowledge process

Based on the object and process view of knowledge management, two key characteristics of knowledge management capability are knowledge stock and knowledge process (Hsu and Shen, 2005; Roper and Hewitt-Dundas, 2015).

IT capabilities can assist organizations in acquiring knowledge more efficiently. By utilizing search engines, databases, and online resources, organizations can quickly access a wealth of knowledge. IT supports data mining and knowledge discovery, enabling valuable knowledge to be extracted from large datasets (Dong and Yang, 2019). The sources of knowledge storage are expanded. Additionally, IT capabilities can also enhance knowledge storage and organization within firms, through the use of knowledge management systems, such as digital archiving systems and advanced search functionality. Organizations can categorize, tag, and index knowledge, making it easier to store and retrieve. Furthermore, cloud storage and virtualization technologies offer increased storage space and flexibility, further facilitating knowledge storage (Smith et al., 2005). Based on this, the following research hypothesis is proposed:

H1a: IT capability has a positive effect on knowledge stock.

IT capabilities facilitate the integration and sharing of knowledge among different departments and systems. By establishing data interfaces and integrated platforms, the problem of knowledge silos can be avoided, enhancing the coherence and consistency of the knowledge process flow (Braojos et al., 2020). IT capabilities enable organizations to have virtual collaboration tools, video conferencing, and online communication platforms, enabling collaboration and knowledge exchange across different geographical locations. This enhances the flow of knowledge among different teams and departments (Ravichandran and Giura, 2019). In addition, IT capabilities can provide support for security and risk management. Through network security technologies, access control, and encryption techniques, security threats and risks in the knowledge flow process can be effectively mitigated (Zhang C. et al., 2023). Based on this, the following research hypothesis is proposed:

H1b: IT capability has a positive effect on the knowledge process.

### 2.4 Knowledge stock, knowledge process capability, and firm performance

Knowledge stock refers to a certain stage within an organization of knowledge resource occupies total. The knowledge are attached to the personnel, equipment, and organizational structure within the organization or system. It is a result of “learning” and reflects the ability and potential for the knowledge production of an organization system (Wu and Shanley, 2009; Roper and Hewitt-Dundas, 2015). The knowledge process relates to the knowledge creation of individuals and organizations (Abubakar et al., 2019). The knowledge process can be defined as the process by which knowledge is created, stored by a person or organization, and shared among members of the organization. Organizations have to pursue research, develop departments, and hire researchers to create new knowledge. The new knowledge can be created through cooperation between the business cooperators (Abubakar et al., 2019).

Knowledge stock, including the accumulated experience, technology, and best practices within an organization, plays a crucial role in providing guidance and direction to the firm (Smith et al., 2005). It reduces the cost of learning and trial and error, enabling faster problem-solving, minimizing errors and redundant efforts, and saving time and resources, ultimately enhancing productivity. Knowledge stock serves as a foundation and reference point for innovation, enabling organizations to continuously seek more efficient approaches. Building upon existing knowledge, new ideas, methods, or processes can be proposed to improve production processes and increase efficiency (Roper and Hewitt-Dundas, 2015). By leveraging knowledge stock, organizations can tap into a wealth of information and insights, reducing the time and effort required to overcome challenges and make informed decisions. This, in turn, leads to improved productivity (Abubakar et al., 2019). Based on this, the following research hypothesis is proposed:

H2a: Knowledge stock has a positive effect on productivity.

An abundant knowledge stock can help businesses develop innovative products or services that differentiate them from competitors. This allows firms to gain a competitive advantage in the market (Roper and Hewitt-Dundas, 2015), leading to increased sales and profitability. Knowledge stock enables businesses to better understand and address challenges and issues within their operational processes, resulting in lower production costs and improved profit margins. By leveraging existing knowledge and experience, firms can identify and mitigate risks more effectively, avoiding costly mistakes and decisions that could negatively impact profitability (Yongping et al., 2011). In addition, a rich knowledge stock helps businesses gain a deeper understanding of market demands and customer preferences, enabling them to conduct targeted marketing activities. By providing products and services that align with customer needs, firms can enhance customer satisfaction and loyalty (Tanriverdi, 2005), ultimately increasing profitability. Based on this, the following research hypothesis is proposed:

H2b: Knowledge stock has a positive effect on profitability.

The knowledge process fosters innovation as different departments and teams can share and exchange knowledge, inspiring and sparking innovative ideas that help improve products, processes, and services (Dong and Yang, 2019), ultimately enhancing productivity. Moreover, knowledge process flow enables the dissemination of best practices, leading to increased productivity. When knowledge freely flows, employees can better leverage each other’s knowledge and experience, avoid redundant work, and enhance work efficiency and quality. Knowledge flow is not only limited to internal operations but can also include acquiring knowledge from external sources. By sharing and exchanging knowledge with partners, suppliers, customers, and industry organizations (Trantopoulos et al., 2017), businesses can gain fresh perspectives and new technologies and ultimately improve productivity. Based on this, the following research hypothesis is proposed:

H2c: Knowledge process has a positive effect on productivity.

The knowledge process flow helps businesses acquire new ideas, technologies, and market insights, thereby promoting innovation. This enables firms to create products or services that differentiate them from competitors, enhancing market competitiveness and profitability (Dong and Yang, 2019). Additionally, by providing timely solutions that meet customer needs, firms can increase profit margins. Similarly, when businesses possess more knowledge and can exchange it with each other, they can avoid redundant work, improve work efficiency, reduce costs, and increase profit margins (Tanriverdi, 2005). Furthermore, during the process of knowledge flow, firms can better identify and address potential risks, making wiser decisions (Hock-Doepgen et al., 2021). This helps in mitigating risks and protecting profit margins. Based on this, the following research hypothesis is proposed:

H2d: Knowledge process has a positive effect on profitability.

According to H0a, H0b, H1a, H1b, H2a, H2b, H2c, and H2d, we suppose that there is a mediating role of knowledge management capability (stock and process) in the relationship between IT capability and firm performance. So we hypothesized as follows:

H3a: Knowledge stock plays a mediating role in the relationship between IT capability and firm performance.

H3b: Knowledge process plays a mediating role in the relationship between IT capability and firm performance.

### 2.5 The moderating role of organizational culture management

Organizational culture management refers to the systematic approach and strategies employed within an organization to shape and maintain specific cultural values, beliefs, behavioral norms, and work environments (Mingaleva et al., 2022). It is widely believed that organizational culture provides the foundation for effective knowledge management and organizational learning (Choo et al., 2008). The literature suggests that cultural management influences knowledge management and sharing practices (De Long and Fahey, 2000; McDermott and O’Dell, 2001; King, 2006; Mueller, 2012). Organizational culture management can improve knowledge capability by influencing personal behavior, awareness, opinions, and beliefs, as well as facilitating the utilization, sharing, and creation of knowledge (Upadhyay and Kumar, 2020).

Organizational culture management highlights the importance of continuous learning and development among individuals and teams, facilitating through training, workshops, and other educational opportunities (Ajmal and Helo, 2010). Such initiatives enable employees to refresh and expand their knowledge and skills based on the accumulation of internal knowledge, thereby offering fresh perspectives for innovation and the enhancement of existing processes (Forman and van Zeebroeck, 2019). The higher the degree of emphasis placed on organizational culture management, the greater the encouragement for innovative thinking and experimentation. Organizations that foster an environment where employees are motivated to explore new methods, technologies, or products are crucial in driving knowledge innovation and application (Mingaleva et al., 2022). This culture promotes the deepening and broadening of knowledge, fostering process improvements among others, which, in turn, contributes to enhanced productivity within the firm. Based on this, the following research hypothesis is proposed:

H4a: Organizational culture management plays a positive moderating role between knowledge stock and firm productivity.

Organizational culture management cultivates an atmosphere that encourages innovative thinking and tolerates failure, thereby creating an environment that inspires employees to explore new ideas, products, and services (Duc et al., 2019). This culture supports leveraging the vast repository of knowledge to develop innovative offerings that differentiate from competitors, enhancing market competitiveness, and subsequently increasing the firm’s profitability. By promoting knowledge sharing and continuous learning, organizational culture management aids employees in better understanding and addressing the complexities and challenges within business processes. This comprehension and approach to problem-solving can reduce production costs and enhance operational efficiency (Bock et al., 2012), directly boosting the firm’s profit margins. Moreover, under a high level of organizational culture management, there is the establishment and maintenance of a positive sense of corporate social responsibility and brand image (Wiengarten et al., 2017). The rich stockpile of knowledge enables more precise marketing strategies. A positive brand image aids in attracting and retaining customers, facilitating deeper marketing engagements and, thus, enhancing the firm’s profitability. Based on this, the following research hypothesis is proposed:

H4b: Organizational culture management plays a positive moderating role between knowledge stock and firm profitability.

The enhancement of organizational culture management significantly contributes to establishing an environment that advocates for open communication and the sharing of knowledge. This cultural framework encourages the flow of knowledge across departments and teams, creating a willingness among employees to share their expertise and experiences. When knowledge circulates freely within an organization, it sparks new ideas and innovation (Bock et al., 2012), thereby elevating the quality of products, processes, and services and consequently enhancing the productivity of the firm. Organizational culture management, by advocating for team diversity and interdisciplinary collaboration, further facilitates the flow of knowledge process (Mingaleva et al., 2022). This strategic approach ensures that employees from varied backgrounds and areas of expertise can come together to share their knowledge and understanding. The integration of diverse thought processes accelerates problem-solving, fosters a more extensive and innovative application of knowledge, and ultimately increases the productivity of the organization. Based on this, the following research hypothesis is proposed:

H4c: Organizational culture management plays a positive moderating role between knowledge process and firm productivity.

Organizational culture management plays a pivotal role in fostering an innovation-driven culture. Within this cultural setting, employees are more inclined to share innovative ideas and technologies, ensuring a smoother flow of knowledge (Ajmal and Helo, 2010). This facilitates the development of unique products and services, enhancing market competitiveness and ultimately leading to an increase in profit margins. Furthermore, organizational culture management aids in the establishment of incentive mechanisms that promote knowledge sharing (Chirico and Nordqvist, 2010). Well-defined incentive mechanisms reward knowledge sharing and team collaboration, enhancing the flow of knowledge. Minimizing the wastage of time and resources directly reduces costs and increases the profit margins of an organization. A higher level of organizational culture management encourages the exchange and sharing of critical information regarding market changes, competitor dynamics, and more. This supports more informed decision-making, safeguarding and enhancing profit margins. Based on this, the following research hypothesis is proposed:

H4d: Organizational culture management plays a positive moderating role between knowledge process and firm profitability.

**Figure 1:**
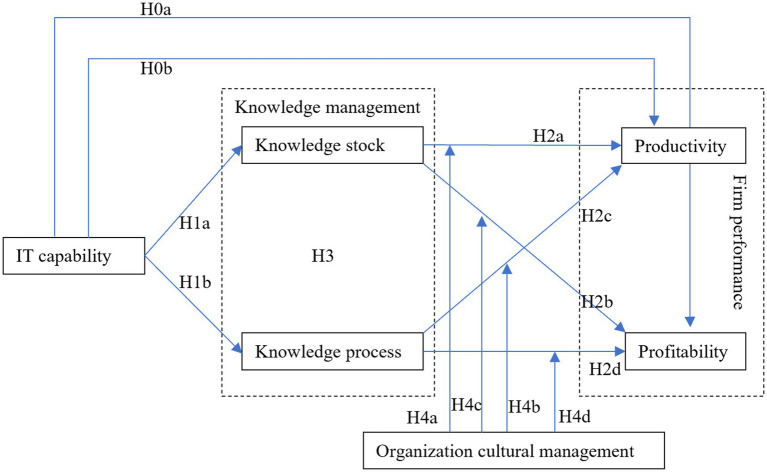
Research framework.

According to the hypothesis development from the previous paragraph, this study has constructed the research framework as follows in Figure 1.

## 3 Methodology

### 3.1 Data and sample

This study targets senior managers in manufacturing firms that have adopted information technology. A total of 200 questionnaires were distributed, and 152 valid questionnaires were recovered, resulting in a valid recovery rate of 76%. The sample details are as follows: As for the firm size, there were 56 firms (36.8%) with fewer than 300 employees, 40 firms (26.3%) with 300–1,000 employees, 44 firms (28.9%) with 1,000–2,000 employees, and 12 firms (7.9%) with more than 2000 employees. Regarding the age of the firms, there are 2 firms (1.3%) that are less than 1 year in operation, 57 firms (37.5%) that have been operating for 1–5 years, 63 firms (41.4%) with an operational span of 5–10 years, and 30 firms (19.7%) that have been in business for over 10 years. As for industry types, there are 45 firms (29.6%) in electronic device manufacturing, 8 firms (5.2%) in the material industry, 30 firms (19.7%) in automobile manufacturing, 12 firms (7.9%) in the electronic industry, 34 firms (22.4%) in software and hardware manufacturing, and 23 firms (15.1%) in the electronic appliances industry.

### 3.2 Measures

According to the conceptual framework, this research examines the correlation between IT capability, knowledge stock, knowledge process, firm performance, and organizational culture management. The content categories are as follows. The score exchange is 5 = strongly agree, 3 = neutral, and 1 = strongly disagree. The measurements of variables are presented in Table 1.

**Table 1:** Construct and survey items.

Variables	Items		Source
Information technology capability (ITC)	Level of informational network		Byrd and Turner (2000) and Ravichandran et al. (2005)
	Number of technical l archives		
	The extent to which technological archives can meet the needs of R&D		
Knowledge stock (KS)	People capability (PC)	The number of R&D staff	OECD (1996), Wu and Shanley (2009), and Roper and Hewitt-Dundas (2015)
		The number of senior technical staff	
		Age structure of R&D staff	
		Level of knowledge	
	Equipment capability (EC)	Completeness of equipment	
		Number of international advanced equipment	
Knowledge process (KP)	Knowledge creation (KC)	Firm has processes for generating new knowledge from existing knowledge	Alavi and Leidner (2001) and Gold et al. (2001)
		Firm has processes for acquiring knowledge about our customers	
		Firm has processes for acquiring knowledge about our suppliers	
	Knowledge sharing (KS)	Firm has processes for distributing knowledge throughout the organization	
		Firm has processes for exchanging knowledge with our business partner	
		Firm has processes for exchanging knowledge between individuals	
		Firm has processes for sharing different sources and types of knowledge	
Organizational culture management (CUL)	Firm culture focuses on technological changes		Cameron and Quinn (2011) and Mingaleva et al. (2022)
	Firm culture is consistent with technological strategy		
	Timely removal of barriers to an innovative culture		
	Recognizes the role of corporate culture in fostering technology development		
Firm performance (PROD)	Profitability		Bottazzi et al. (2008)
	Productivity		

In the initial data collection of this study, there were many items of variables based on reference. However, due to the small factor load of some items, a few items were eventually deleted, and only the items used in the final study are presented in this table.

Several control variables were used in this study. In this study, we controlled three demographic variables that were of no direct interest but were related to the work outcome, and the control variables in this study included firm size, firm age, and type of industry (Rai et al., 2015; Yin et al., 2020). We measured the firm size as the total number of employees in the firm, and we measured the firm age as the number of years that the firm has been established; we considered firm type as a high/low technology as control variables.

## 4 Results

Table 2 lists descriptive statistics, including mean, standard deviation, and correlation. It clearly shows that productivity and profitability have a significantly positive correlation with IT capability, people capability, equipment capability, knowledge creation, knowledge process, and organizational culture. The correlation coefficients of variables are significantly higher than 10%.

**Table 2:** Descriptive statistics.

	Mean	SD	1	2	3	4	5	6	7
1. ITC	3.462	0.508	1						
2. PC	3.615	0.352	0.455**	1					
3. EC	3.798	0.545	0.462**	0.533**	1				
4. KC	3.494	0.464	0.299*	0.425**	0.441**	1			
5. KS	3.490	0.440	0.232**	0.356**	0.268**	0.336*	1		
6. CUL	3.476	0.365	0.326**	0.490**	0.345*	0.371*	0.487**	1	
7. PROD	3.827	0.585	0.164**	0.147**	0.134**	0.152**	0.127**	0.325**	1
8. PROF	3.885	0.646	0.325**	0.254**	0.239**	0.241**	0.210**	0.342**	0.413**

*P < 0.1. **p < 0.05. ***p < 0.01.

This research conducted a confirmatory factor analysis for validity testing. The factor loadings for various items ranged from 0.612 to 0.755, all exceeding the threshold value of 0.500. The average variance extracted (AVE) values were all above 0.400, and the composite reliability (CR) values were all above 0.700 (as detailed in Table 3), indicating that the questionnaire has good validity. Additionally, reliability testing was performed using Cronbach’s alpha, with all values exceeding the threshold of 0.700, which suggests that the questionnaire is reliable (as shown in Table 3).

**Table 3:** Reliability and validity analysis.

	Alpha	AVE	CR
1. ITC	0.754	0.450	0.710
2. PC	0.761	0.420	0.743
3. EC	0.724	0.545	0.705
4. KC	0.711	0.462	0.720
5. KS	0.702	0.466	0.776
6. CUL	0.773	0.410	0.736

The results in Table 4 presented the results of hypothesis testing. The result from the table above showed that, in model 1, the IT capability has a positive effect on productivity (*β =* 0.38, *p* < 0.01), and the result of model 2 showed that the IT capability has a positive effect on profitability (*β =* 0.39, *p* < 0.01). H0a and H0b are supported. The above table in model 3 shows that IT capability has a positive effect on knowledge stock (*β =* 0.33, *p* < 0.01). The result supported H1a, which states that IT capability has a positive effect on stock, and the result of model 4 from the table also showed that IT capability determines the knowledge process (*β =* 0.46, *p* < 0.01). The result supported H1b, which states that IT capability has a positive effect on the knowledge process. Model 5 showed that knowledge stock has a positive effect on productivity (H2a) (*β =* 0.27, *p* < 0.01), followed by model 6, which showed that knowledge stock has a positive effect on profitability (H2b) (*β =* 0.30, *p* < 0.01). Thus, hypotheses H2a and H2b were supported. Model 7 showed that the knowledge process has a positive effect on productivity (H2c) (*β =* 0.21, *p* < 0.01), followed by model 8, which showed that the knowledge process has a positive effect on profitability (H2d) (*β =* 0.17, *p* < 0.01). Thus, hypotheses H2c and H2d were supported. Based on the results, including H0a, H0b, H1a, H2b, H2a, H2b, H2c, and H2d, we conclude that knowledge management capability (stock and process) plays a positive mediating role between IT capability and firm performance. As a result, H3a and H3b were supported.

**Table 4:** The result of regression analysis.

	PROD	PROF	KS	KP	PROD	PROF	PROD	PROF	PROD	PROF
	Model 1	Model 2	Model 3	Model 4	Model 5	Model 6	Model 7	Model 8	Model 9	Model 10
FS	0.23	0.21	0.13	0.14	0.15	0.13	0.23	0.21	0.11	0.14
FA	0.31	0.11	0.06	0.06	0.12	0.20	0.06	0.15	0.12	0.07
TOI	0.14	0.11	0.02	0.18	0.179	0.15	0.13	0.12	0.07	0.17
ITC	0.38***	0.39***	0.33***	0.46***	0.29***	0.27***	0.26***	0.28***	0.23***	0.25***
KS					0.27***	0.30***			0.33***	0.32***
KP							0.21***	0.17***	0.31***	0.26***
CUL									0.21***	0.19***
KS*CUL									0.18***	0.16***
KP*CUL									0.17***	0.12***
R^2^	0.25	0.23	0.26	0.31	0.26	0.26	0.28	0.29	0.30	0.31
Adj R^2^	0.19	0.18	0.21	0.26	0.19	0.19	0.22	0.22	0.18	0.19
F	4.75***	4.26***	4.92***	6.29***	3.94***	3.94***	4.36***	4.57***	2.47***	2.59***
Obs.	152	152	152	152	152	152	152	152	152	152

*P < 0.1. **p < 0.05. ***p < 0.01.

The results of model 9 and model 10 from Table 4 showed the moderating effect of organizational culture management. From model 9, the organizational culture management moderated the relationship between knowledge stock and productivity (*β =* 0.18, *p* < 0.01) and moderated the relationship between the knowledge process and productivity (*β =* 0.17, *p* < 0.01). Thus, H4a and H4b were supported. The result of model 10 showed that the organizational culture management moderated the relationship between knowledge stock and profitability (*β =* 0.16, *p* < 0.01) and it also moderated the relationship between the knowledge process and profitability by (*β =* 0.12, *p* < 0.01). Thus, H4c and H4d were supported.

## 5 Discussion and conclusion

From the results, based on the positive relationship between IT capability and performance (productivity and profitability), we found that IT capability has a positive effect on knowledge stock/process, and knowledge stock/process has a positive effect on productivity/profitability, which show the partial mediating effects of knowledge management capability between IT capability and firm performance. IT capability enhances knowledge stocks by expanding the sources of data and effectively storing knowledge. It affects knowledge processes by facilitating knowledge dissemination and sharing, as well as fostering knowledge transfer and collaboration. A rich stock of knowledge can improve a firm’s decision-making efficiency, innovation ability, problem-solving capacity, and business process optimization, thereby enhancing the firm’s productivity. Through advancements in innovation, cost control, and market insights, a substantial knowledge base can lead to increased sales, reduced costs, and improved customer satisfaction, thus elevating profitability. The knowledge process improves work efficiency, problem-solving ability, and continuous improvement capability through knowledge sharing, collaboration, decision support, learning, and innovation, thereby enhancing productivity. By boosting innovation ability, cost control, market insights, and risk management, the knowledge process can increase sales, reduce costs, and enhance customer satisfaction, ultimately leading to higher profit margins.

Furthermore, we also found the moderating effects of organizational culture management. Organizational culture management has a positive moderating effect on the relationship between knowledge management capability (knowledge stock and process) and firm performance (productivity and profitability). Organizational culture management plays a moderating role by encouraging cross-border cooperation and diverse thinking, organizational learning and innovative cultural construction, and creating an open and shared cultural environment.

### 5.1 Theoretical implications

First, our finding contributes to the IT capability literature by the mediating mechanism of IT capabilities on firm performance. Contrary to the perspectives in recent research that IT capabilities have a negative impact or no effect on firm performance (Chae et al., 2014; Rahman and Zhao, 2020; Mamonov and Peterson, 2021), our examination still substantiates the positive role of the former (Hao and Song, 2016; Li and Chan, 2019). It is supported that those findings still hold true after decades of dramatic change in the business use of technologies, especially the growing popularity and application of IT as part of business strategy. Additionally, it demonstrates that knowledge management capabilities play a partial mediating role, validating a novel “Capability-Knowledge-Performance” mechanism. This also responds to the call for further exploration of the mediating mechanisms between IT capabilities and firm performance.

Second, this study contributes to the research of knowledge management theory. This study, based on the process and object view of knowledge (Hsu and Shen, 2005; Roper and Hewitt-Dundas, 2015), categorizes knowledge management into knowledge stock and knowledge process flows. It explores the mechanisms and pathways for improving firm performance. We find that IT capabilities positively impact both, and both have a positive effect on firm performance. Knowledge stock and knowledge process partially mediate the relationship between IT capabilities and firm performance, which enriches the research on different perspectives of knowledge management with a more detailed exploration of the role of knowledge in the process of transforming resources into performance (Xie et al., 2016; Abubakar et al., 2019).

Third, this study contributes to the research on organizational culture management. We make a theoretical contribution by examining the moderating role of organizational culture management and enriching the literature on culture management and knowledge management capabilities (Chen et al., 2015; Wang et al., 2023). We found that organizational culture management played a positive moderating role in the relationship between knowledge management capability and firm performance, that is, if the firm has a strong organizational culture, it can help the firm reach a high level of performance by joint knowledge management capability. This enriches institutional theory and knowledge management theory.

### 5.2 Managerial implications

Our research has important managerial implications. First, the firm manager should encourage IT resources and use them effectively to gain good IT capability because IT capability can improve firm performance, and the manager should recognize that IT resources and IT capability are important to improve firm performance. Second, we would like to give advice to the manager of the company that the manager should continuously enhance IT capability and knowledge capability to enhance organizational performance, and the manager should pay attention to the development of IT capability and knowledge capability as the important factors that influence organizational performance. Third, we propose to promote the firm’s organizational culture management. Furthermore, the cultivation of the right organizational culture to encourage knowledge capability in an organization can enhance firm performance. Managers should pay attention to organizational culture management, construct an innovative culture, knowledge-sharing culture, and learning culture, as well as encourage systems.

### 5.3 Research limitation and future research

There were limitations in this study. In this dissertation, we studied the relationship between IT capability, knowledge management capability, firm performance, and organizational culture management in the questionnaires. Since the sample size itself is relatively small and regional, there are limitations in a deeper understanding of the relationship between IT and firm performance. In the future, this study can be strengthened by increasing the different samples from different countries and industries. Continuing studies can try to continuously track certain firms to study the impact of time effects. Furthermore, one can try to explore the role or differences of specific IT technologies, as well as other new potential mediating or moderating mechanisms between IT capability and firm performance in the digital era. Future research may further explore how different types of IT capabilities influence specific types of business performance through various knowledge management processes or how they operate within different organizational cultural contexts.

## Data availability statement

The raw data supporting the conclusions of this article will be made available by the authors, without undue reservation.

## Author contributions

WW: Writing – original draft, Writing – review & editing, Funding acquisition, Resources. XL: Writing – original draft, Writing – review & editing, Conceptualization, Formal analysis, Methodology, Software. BS: Formal analysis, Supervision, Validation, Visualization, Writing – original draft, Writing – review & editing.
